# Análisis de costo-efectividad del uso del programa VECTOS en el control rutinario de enfermedades transmitidas por *Aedes aegypti* en dos municipios de Santander, Colombia

**DOI:** 10.7705/biomedica.4658

**Published:** 2020-06-30

**Authors:** Manuel Alejandro Salinas, Victoria Eugenia Soto, Sergio Iván Prada

**Affiliations:** 1 Centro de Estudios en Protección Social y Economía de la Salud, PROESA, Universidad Icesi, Cali, Colombia Universidad Icesi Centro de Estudios en Protección Social y Economía de la Salud, PROESA Universidad Icesi Cali Colombia

**Keywords:** Aedes aegypti, dengue, control de vectores, análisis costo-beneficio, programas informáticos., Aedes aegypti, dengue, vector control, cost-benefit analysis, software.

## Abstract

**Introducción.:**

Las enfermedades transmitidas por *Aedes aegypti* son un problema de salud pública. VECTOS es un programa novedoso de integración de estrategias de control de vectores.

**Objetivo.:**

Evaluar el costo-efectividad del uso del VECTOS en los programas de control rutinario de enfermedades transmitidas por el vector *Aedes aegypti* en el municipio de San Juan de Girón (Santander).

**Materiales y métodos.:**

Se evaluó el costo-efectividad del programa empleando un modelo de análisis de decisiones desde la perspectiva de las autoridades locales de salud. Se estudió la integración de las estrategias de control de vectores mediante el programa VECTOS utilizado en el municipio de San Juan de Girón durante el 2016, con el control rutinario llevado a cabo sin VECTOS en el municipio de Floridablanca. Se calculó la razón incremental del costo-efectividad (RICE), usando como medida de efectividad los años de vida ajustados por discapacidad (AVAD).

**Resultados.:**

El uso del programa VECTOS fue rentable a una tasa de ahorro de USD$ 660,4 por cada AVAD evitado en comparación con el control de rutina en Floridablanca. El modelo probabilístico indicó que el sistema fue costo-efectivo en el 70 % de las 10.000 iteraciones para un umbral entre 1 y 3 PIB per cápita.

**Conclusiones.:**

El programa VECTOS fue muy costo-efectivo en el municipio de San Juan de Girón. Su uso puede adoptarse en otros municipios del país donde las enfermedades transmitidas por *A. aegypti* son endémicas.

## Abtract

**Introduction:**

Diseases transmitted by *Aedes aegypti* are considered a public health problem. VECTOS is a novel software for the integration of vector control strategies. Objective: To assess the cost-effectiveness of the use of VECTOS in the routine control programs of diseases transmitted by *A. aegypti* in the municipality of San Juan de Girón (Santander).

**Materials and methods:**

We conducted a cost-effectiveness analysis using a decision analysis model from the perspective of the local health authorities. We considered the use of the VECTOS software in the routine control activities in the municipality of San Juan de Girón during 2016 as the treatment group while the routine control in the municipality of Floridablanca, where VECTOS is not implemented, was considered as the comparator. We calculated the incremental cost-effectiveness ratio (ICER) taking as effectiveness measure the disability-adjusted life years (DALY).

**Results:**

VECTOS was cost-effective at a rate of USD$ 660,4 savings per each DALY avoided compared to the routine control in Floridablanca. The probabilistic model showed that the system was cost-effective in 70% of the 10.000 iterations for a threshold between 1 to 3 GDP per capita.

**Conclusions:**

VECTOS software as implemented in the municipality of San Juan de Girón is highly cost-effective and could be used in other municipalities in the country where diseases transmitted by *A. aegypti* are endemic.

*Aedes aegypti* es el principal vector transmisor de dengue, Zika y chikungunya en Colombia. Estas enfermedades representan un grave problema de salud pública en el país; en el 2016, cerca de 103.822 personas contrajeron el virus del dengue ^1^ y, en el 2010, 151.774 personas, siendo este el peor año en la última década ^2^. Cerca del 55 % de la población colombiana se encuentra en riesgo de contraer estas enfermedades o morir a causa de ellas, pues *A. aegypti* está presente en zonas que se encuentran por debajo de los 2.200 msnm ^3^.

En términos económicos, el costo del dengue en el país se cuantificó, aproximadamente en USD$167 millones en el 2010, con un registro de 292 muertes confirmadas en ese año y una carga estimada en 57.017 años de vida ajustados por discapacidad (AVAD) ^4^.

El Centro Internacional de Entrenamiento e Investigaciones Médicas (CIDEIM), junto con la Corporación para la Investigación de la Corrosión (CIC) y las secretarías de salud de San Juan de Girón (Santander), Yopal (Casanare) y Buga (Valle), desarrollaron un sistema integrado de gestión cuyo producto principal es el programa llamado VECTOS.

Este programa permite visualizar la información epidemiológica, entomológica y social georreferenciada diariamente, así como emitir alertas tempranas por barrio y hacer un análisis estratificado del riesgo según los índices de infestación, los casos reportados y el número de criaderos, entre otras variables. Dicha información busca responder a los diversos factores determinantes de transmisión del dengue en los contextos locales mediante el análisis y la evaluación de las variables epidemiológicas, entomológicas y sociales asociadas con su transmisión, con el fin de facilitar la toma de decisiones informadas y, así, facilitar el diseño de estrategias de prevención y control del dengue.

El objetivo de este estudio fue analizar el costo-efectividad del programa VECTOS, diseñado para integrar las estrategias de control rutinario de vectores, en el municipio de San Juan de Girón (Santander) durante el 2016. Este programa se comparó con el control rutinario del municipio de Floridablanca, de características similares a las de Girón, en donde no se usó VECTOS, pero también se planean y ejecutan las estrategias de control vectorial según las directrices de las guías del Instituto Nacional de Salud.

## Materiales y métodos

En los estudios de costo-efectividad, es usual establecer la pregunta de evaluación económica mediante la estrategia denominada PICOT (población, intervención, control, *outcome* o resultado y tiempo) ^5^. A continuación, se define cada uno de los elementos empleados en la pregunta de evaluación económica.

### Población

VECTOS se diseñó para las cabeceras urbanas de los municipios donde el dengue es una enfermedad endémica. En este estudio, en particular, se empleó la información recolectada en Girón, municipio donde se ha usado el programa VECTOS desde el 2015. San Juan de Girón se ubica en el departamento de Santander y su cabecera urbana tenía 170.917 habitantes en el 2016 ^6^.

### Perspectiva del análisis

Se adoptó la perspectiva del municipio, dado que VECTOS está concebido como un sistema de planeación y focalización local. La autoridad de salud local es responsable de reducir la morbilidad de las enfermedades transmitidas por *A. aegypti* y la disponibilidad local de recursos es limitada. En este marco, los costos que se deben tener en cuenta son los empleados por las secretarías de salud de los entes territoriales en la planeación y ejecución de sus programas de control vectorial.

### Intervención

VECTOS es un programa integrado de análisis y estratificación de las variables epidemiológicas, entomológicas y sociales asociadas con la transmisión de dengue. El programa facilita la adopción de decisiones informadas mediante la visualización georreferenciada de la información recolectada diariamente, con una interfaz que permite emitir alertas tempranas por barrio y hacer el análisis estratificado del riesgo, según los índices de infestación, los casos reportados y el número de criaderos, entre otras variables. Ello contribuye al diseño y evaluación de las estrategias de prevención y control de las enfermedades transmitidas por *A. aegypti* en contextos locales.

El programa integra esta información y genera indicadores de estratificación del riesgo para el barrio, visualizándolos de forma georreferenciada en tiempo real, lo que permite a las autoridades adoptar estrategias de control focalizadas en los barrios o comunas y según los factores de riesgo preponderantes en cada localidad.

La información entomológica es recolectada mediante la aplicación móvil SPECTRA que está integrada con VECTOS y permite el ingreso directo de los datos al sistema por parte de los técnicos del municipio encargados de los levantamientos entomológicos. La aplicación tiene en cuenta la productividad de los mosquitos por hogar mediante el conteo de pupas y la detección de larvas en los recipientes de agua (índice de Breteau). Además, caracteriza los contenedores de agua en los hogares para determinar los factores de riesgo en el hogar y extrapolarlos a la unidad espacial del barrio.

El programa capta los datos epidemiológicos suministrados al Sistema Nacional de Vigilancia en Salud Pública (Sivigila). Las secretarías municipales de salud tienen la función de recolectar el reporte de los casos de interés en salud pública entregado por los prestadores de servicios de salud, información que es compilada e ingresada semanalmente al Sivigila y al sistema VECTOS, el cual se encarga de depurar la información, caracterizarla por caso y georreferenciarla por barrio con base en la dirección reportada. El componente social del sistema obtiene información de las encuestas realizadas por los técnicos en la visita a los hogares. La encuesta busca capturar información sobre las condiciones socioeconómicas de riesgo, como estado de la vivienda, acceso a los servicios públicos y hacinamiento, entre otros.

Una vez integrados estos componentes, el programa estratifica el riesgo por barrio y sugiere qué estrategias se deben implementar en cada uno, con base en los factores de riesgo de transmisión. Asimismo, prioriza las estrategias de control implementadas en el municipio según si el riesgo proviene de la presencia de criaderos, potenciales criaderos y vectores adultos, de la incidencia de enfermedades o de las condiciones sociales de los hogares. Las estrategias implementadas en ambos municipios fueron las recomendadas en la guía del Instituto Nacional de Salud y el Ministerio de Salud y Protección Social, las cuales incluyen la eliminación de criaderos, la intervención de focos específicos (por ejemplo, tanques altos y sumideros) y el control químico del vector en la etapa larval y la adulta, incluida la entrega de mosquiteros tratados con insecticida en Girón. También, se hicieron campañas educativas e informativas de prevención en barrios e instituciones prestadoras de servicios de salud (IPS), y a poblaciones vulnerables.

El programa incluye una interfaz intuitiva y de fácil manejo; está diseñado para ser usado por los coordinadores de los programas de enfermedades transmitidas por vectores (ETV) de los municipios y tiene un módulo de consulta que los secretarios de salud o el personal involucrado en las estrategias de control de las enfermedades transmitidas por vectores pueden usar.

### Control

VECTOS se comparó con el programa rutinario de control vectorial de Floridablanca, donde no se usó VECTOS, pero sí se utilizaron las estrategias de control de las enfermedades transmitidas por vectores contempladas en las guías del Instituto y la información entomológica y epidemiológica que se recolecta localmente. Este municipio, a 1 km de Bucaramanga, limita con Girón y, al igual que este, hace parte del área metropolitana de la ciudad. Su población proyectada en cabeceras urbanas es de 257.265 habitantes ^7^.

**Figura 1:**
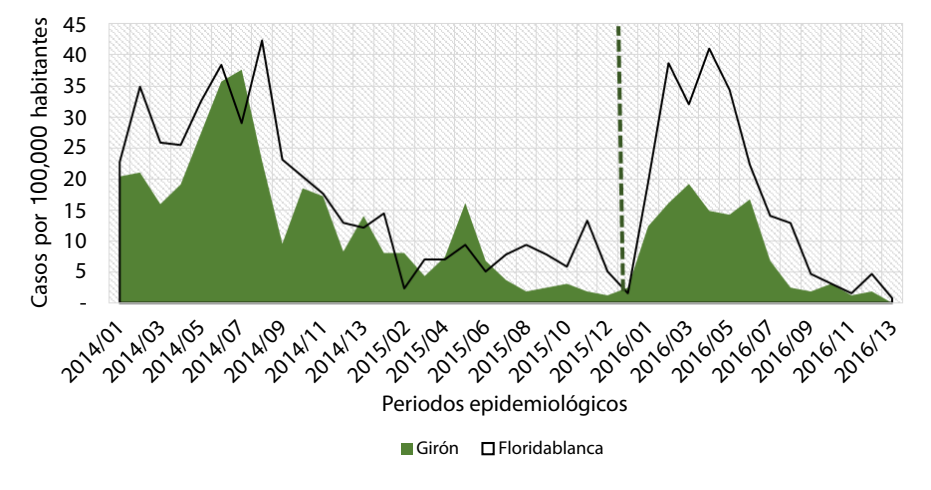
Incidencia del dengue (2014-2016) en el municipio de Girón (área verde) intervenido con el programa VECTOS e intervenciones rutinarias de control en el municipio de Floridablanca (línea negra, control). La línea vertical punteada indica el mes de enero de 2016, cuando se empezó a usar VECTOS en el municipio de Girón. Fuente de datos: Sivigila

En la figura 1 se ilustra el comportamiento temporal de la incidencia del dengue por cien mil habitantes en los dos municipios evaluados, aparentemente con un alto grado de correlación entre ellos en el periodo de enero de 2014 a diciembre de 2016 (coeficiente de correlación de Pearson de 0,802). En cuanto a la magnitud de la incidencia, en el periodo entre el 2014 y el 2015, el comportamiento de las incidencias fue similar, con un nivel de confianza del 95 % (t=1,8709, gl=25) (p=0,07), en tanto que, durante el 2016, se presentó una divergencia entre febrero y mayo, siendo la incidencia significativamente mayor en el municipio de comparación, con un nivel de confianza del 95 % (t=3,2736, gl=12) (p=0,007).

### Resultado final

En el estudio se consideraron los AVAD derivados de los casos reportados de dengue y muertes por dengue como resultado final. La ecuación 1 muestra el cálculo empleado para estimar esta variable: AVAD = AVP + AVD, donde AVP es años de vida perdidos y AVD, años vividos con discapacidad.

Para el cálculo de los años de vida perdidos se empleó la ecuación 2:



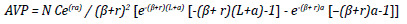



Los años vividos con discapacidad se calcularon mediante la ecuación 3:



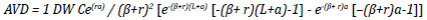



donde para la ecuación, 2 N corresponde al número de muertes por dengue, mientras que para la ecuación 3, I corresponde al número de casos incidentes; DW, al ponderador de discapacidad, y para ambas ecuaciones C, a la constante correctora del ponderador de edades; r, a la tasa de descuento; β, al parámetro de la función ponderadora de edades; a, a la edad de inicio de síntomas, y L, a los días perdidos por discapacidad o por muerte prematura.

No se tuvo en cuenta la información reportada sobre casos de Zika y chikungunya, dado que en Colombia no se dispone de información sobre la carga de estas enfermedades causadas, además, por virus introducidos en el país recientemente. Se emplearon los parámetros estimados en un estudio sobre la carga de enfermedad del dengue para Colombia ^4^. Con el diferencial entre la razón de costos y la efectividad de implementación de VECTOS, y la alternativa de no implementar este programa, se obtuvo la razón incremental de costo-efectividad (RICE) representada por la ecuación 4:



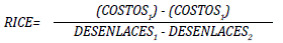



En esta razón, el subíndice 1 denota el uso de VECTOS y, el subíndice 2, el programa de control de vectores rutinario en Floridablanca.

### Horizonte temporal

El horizonte temporal del estudio fue de un año con los datos recolectados para el 2016, con el fin de reflejar los efectos de llevar a cabo estrategias de control vectorial basadas en la evidencia ^8^.

### Costos

Los costos se establecieron con la metodología de microcosteo de abajo hacia arriba, cuantificando los recursos empleados en cada una de las actividades ejecutadas mensualmente ^9^. Se clasificaron por estrategia y costos recurrentes y de capital, según la metodología de la Organización Mundial de la Salud (OMS) ^10^.

La fuente de los datos sobre los costos de las intervenciones provino de la contabilidad de los municipios, la contratación, los registros trimestrales de ejecución de recursos y los registros diarios de ejecución de actividades usados en el CIDEIM.

En esta fase del proyecto, los costos de VECTOS fueron asumidos por el CIDEIM y los de esta evaluación se cargaron al programa de control de vectores de Girón. El costo asignado a VECTOS corresponde al valor mensual cobrado a un municipio por los servicios de implementación, puesta en marcha y acompañamiento continuo en el uso del sistema, e incluye el recurso humano, los insumos y el recurso tecnológico. La totalidad del costo del programa no podía ser asignada a un solo municipio, ya que está concebido como un sistema que integra un número significativo de entes territoriales.

Parte del costo del programa de enfermedades transmitidas por vectores de Girón en este estudio, provino del estudio sobre los costos de los programas de control vectorial para entes territoriales en Colombia de Salinas-López, *et al.*^11^.

Otros costos para los que no se contaba con una fuente confiable, se tomaron de la tabla de precios internacionales disponible en el portal WHO- CHOICE de la OMS ^12^ y se convirtieron a pesos colombianos (COP) del 2016 y a dólares americanos (USD) a una tasa de cambio de COP$ 3.053,42 por USD$ 1, la cual corresponde al promedio anual de la tasa representativa del mercado en el 2016.

### Modelo

Para las evaluaciones económicas de enfermedades infecciosas, se considera más apropiado el uso de modelos dinámicos, ya que ofrecen una aproximación más realista de su transmisión ^13^. En el caso del dengue, un modelado de este tipo requiere parámetros que involucran una interacción compleja entre humanos, vectores, ambiente y serotipos del virus ^14^, información que no estaba disponible para los casos estudiados en los programas rutinarios de control de Girón y de Floridablanca.

Por lo tanto, se consideró el uso de un árbol de decisiones como modelo de análisis y se asumió la transmisión del dengue como estática, por estar capturada únicamente en la tasa de incidencia por cien mil habitantes en cada una de las poblaciones analizadas, lo que refleja las condiciones de incertidumbre y las disyuntivas relevantes que implica la evaluación de un programa de este tipo ^15^.

Ante la ausencia de un umbral en términos de AVAD evitados para Colombia, se tomó como umbral de costo-efectividad el planteado por la comisión macroeconómica de la OMS, la cual estableció que una estrategia es muy costo-efectiva si tiene un valor de un PIB per cápita por AVAD evitado y es costo-efectiva si es inferior a tres veces el PIB per cápita de cada país ^16^.

Los parámetros empleados en la estimación del modelo se presentan en el cuadro 1, y provienen de los datos recolectados y de la literatura especializada. Para la estimación del modelo, se empleó el programa TreeAge Pro 2012™ (TreeAge Software Inc., Williamstown, MA, USA).

**Cuadro 1:** Parámetros del modelo de decisión con los valores, los rangos en que fluctúan los parámetros, las distribuciones de probabilidad y el origen o la fuente de los datos. Los parámetros que acompañan la distribución gamma son κ y λ y los que acompañan la distribución beta son α y β, los cuales se calcularon a partir de una aproximación de la media y la desviación estándar.

Parámetro	VECTOS y control rutinario (Girón)			Control rutinario (Floridablanca)		
	Valor de base	Distribución	Fuente	Valor de base	Distribución	Fuente
	(Rango)			(Rango)		
Tasa de incidencia anual de dengue (%)	0,28	Beta (459; 166,149)	SIVIGILA	0,54	Beta (1,391; 255,315)	SIVIGILA
Peso de discapacidad del dengue	0,81 (0,6-0,92)	Estimacion fija	-4	0,81 (0,6-0,92)	Estimacion fija	-4
Costo promedio mensual de personal (USD)	4.188	Gamma (6,46; 0,00154)	Recoleccion de datos	6.321	Gamma (6,45; 0,00102)	Recoleccion de datos1
Costos operacionales, promedio mensual (USD)	1.833	Gamma (6,45; 0,003)	Recoleccion de datos	3.603	Gamma (6,46; 0,00179)	Recoleccion de datos
Costos de insumos, promedio mensual (USD)	0	-	-	846	Gamma (6,45;0,00763)	Recoleccion de datos
Costos de capital, promedio mensual ($USD)	1.424	Gamma (6,47; 0,00454)	Recoleccion de datos	3.383	Gamma (6,45; 0,00191)	Recoleccion de datos
Costo de VECTOS, promedio mensual (USD)	3.398	Gamma (6,46; 0,0019)	Recoleccion de datos	0	-	-
Costo de toldillos, promedio mensual (USD)	776	Gamma (6,47; 0,00834)	Recoleccion de datos	0	-	-
Costo de larvicida, promedio mensual (USD)	88	Gamma (6,32; 0,07184)	Recoleccion de datos	0	-	No se modelo
Costo de adulticida, promedio mensual (USD)	515	Gamma (6,44; 0,0125)	Recoleccion de datos	0	-	No se modelo
AVAD	17,62 (13,05-20,01)	Estimacion fija	Calculos propios	52,71 (39,05-59,87)	Estimacion fija	Calculos propios
	Umbral de costos (USD$) 5,861-17,583 Analisis de umbral					

^1^ Contabilidad de los municipios, contratación y registros trimestrales de ejecución de recursos, y registro diario de ejecución de actividades del CIDEIM

## Resultados

El análisis de costos de cada uno de los programas de control de las enfermedades transmitidas por vectores de los municipios estudiados, se presenta en el cuadro 2. En general, el programa de Floridablanca fue más costoso que el de Girón, especialmente por los costos recurrentes que representaron el 77 % del total; el rubro de personal representó cerca del 45 % del total. El uso y la implementación de VECTOS para el control rutinario de vectores en Girón, representaron cerca del 28 % del total de los costos, lo que equilibró la composición entre costos recurrentes y de capital del programa. No obstante, sin contar el uso de VECTOS, el programa es intensivo en costos recurrentes, principalmente los de personal.

**Cuadro 2:** Costos de los programas de enfermedades transmitidas por vectores en cada municipio en el 2016 (cifras en USD)

Rubros	VECTOS y control	%	Control rutinario	%
	rutinario (Girón)		(Floridablanca)	
Recurrentes	79.488		129.237	
Personal	50.254	34,3	75.855	44,7
Operacionales	22.001	15	43.230	25,5
Insumos	7.233	4,9	10.153	6
Capital	67.163		40.596	
VECTOS	40.770	27,8	-	0
Capital	26.393	18	40.596	23,9
Total	146.651	100	169.834	100

Los resultados del modelo determinístico sugieren que la alternativa de implementación de VECTOS en Girón fue más costo-efectiva que el programa rutinario de enfermedades transmitidas por vectores ejecutado en Floridablanca. En el cuadro 3, se resumen los resultados del análisis determinístico. Cabe destacar que el programa rutinario de enfermedades transmitidas por vectores de Floridablanca costó USD$ 23.172 más que el de Girón, incluso agregando el costo mensual promedio de USD$ 3.398 atribuible al VECTOS en este último municipio (cuadro 2).

**Cuadro 3:** Estimación del modelo determinístico en términos de la razón incremental de costo- efectividad (RICE)

Alternativas	Costo	Costo incremental	AVAD	AVAD	RICE	Resultado
	(USD)	(USD)		evitados		
VECTOS y control rutinario (Girón)	$ 146.664		17,62			
Control rutinario (Floridablanca)	$ 169.836	$ 23.172	52,71	35,09	$ 660,36	Dominada

AVAD: años de vida ajustados por discapacidad; RICE: razón incremental de costo-efectividad

En cuanto a la efectividad, aunque el programa de Floridablanca destinó más recursos, se observó que la carga del dengue es de 35,09 AVAD más que en Girón, lo que evidencia la poca efectividad de su programa para reducir la morbilidad por dengue comparado con el de Girón. La razón incremental de costo-efectividad (RICE) indicó que, en el control rutinario y el uso de VECTOS en Girón, se emplearon USD$ 660,4 menos por cada AVAD evitado que en el control rutinario de Floridablanca.

Los resultados individuales en el modelo planteado se resumen en el cuadro 4. Estos se obtienen de ingresar al modelo la información de un individuo, teniendo en cuenta sus probabilidades de contraer la enfermedad en cada una de las alternativas del árbol de decisión, e indicaron que seguía siendo más costo-efectivo el uso de VECTOS, pues en Girón se evitaron 0,24 AVAD a un costo inferior a los USD$ 23.586, comparado con el control rutinario de Floridablanca. Cabe anotar que la magnitud del resultado final disminuyó porque, en estos municipios, la probabilidad de que un individuo se infecte es baja y esta probabilidad se derivó de la incidencia anual de dengue en el 2016.

**Cuadro 4:** Estimación del modelo determinístico en términos de la razón incremental de costo- efectividad (RICE) por individuo en el modelo de decisión

Alternativas	Costo	Costo incremental	AVAD	AVAD	RICE	Resultado
	(USD)	(USD)		evitados		
VECTOS y control rutinario (Girón)	$ 146.664		0,05			
Control rutinario (Floridablanca)	$ 169.836	$ 23.586	0,29	0,24	98.033	Dominada

AVAD: años de vida ajustados por discapacidad; RICE: razón incremental de costo-efectividad

El análisis univariado tomando como variable de efectividad los AVAD para el control rutinario más VECTOS en Girón, indicó que el umbral de indiferencia entre ambas alternativas se alcanzó cuando esta variable tomó el valor de 103,5 AVAD, en tanto que el análisis univariado para la variable del costo total ubicó el umbral de indiferencia de costo-efectividad en USD$ 169.836 en el caso del control rutinario de Floridablanca. Los resultados de estos análisis de sensibilidad indican que el costo-efectividad fue mucho más sensible a los cambios en el costo del programa, puesto que el umbral en términos de AVAD equivalió a casi seis veces la medición inicial del programa rutinario más VECTOS en Girón, en tanto que el umbral de costos equivalió a casi el 16 % de la estimación inicial de costos.

**Figura 2:**
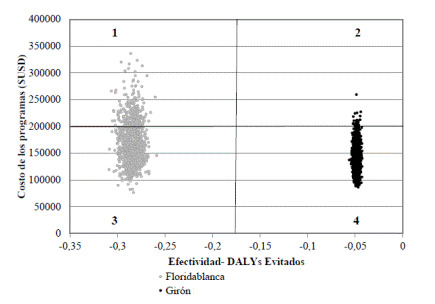
Análisis de sensibilidad, diagrama de dispersión de 10.000 iteraciones del modelo de costo-efectividad. Cuadrante 1: posible situación menos deseable, más costosa y menos efectiva. Cuadrantes 2 y 3: compensación entre ahorro en costos u obtener más efectividad. Cuadrante 4: posible situación más deseable, menos costosa y más efectiva

Los resultados del análisis probabilístico también sugieren que el sistema VECTOS es la alternativa más costo-efectiva. En la figura 2 se presenta el gráfico de dispersión de las 10.000 iteraciones de la simulación de Montecarlo y se observa que la mayoría de los puntos de la alternativa VECTOS se ubican en la zona de mayor efectividad y menor costo, frente a la alternativa del control rutinario de Floridablanca. En promedio, la efectividad de la alternativa VECTOS fue de 0,05 AVAD y su costo promedio fue de USD$ 146.484, en tanto que la efectividad promedio de la alternativa del control rutinario de Floridablanca fue de 0,28 AVAD y el costo promedio fue de USD$ 170.070. La curva de aceptabilidad expresa el porcentaje de iteraciones que resultaron costo-efectivas en los varios niveles de disponibilidad para pagar de las alternativas. Cerca del 70 % de las 10.000 iteraciones de la alternativa VECTOS fue costo-efectivo en un rango de disponibilidad para pagar de 1 a 3 PIB per cápita en Colombia en el 2016.

## Discusión

Los resultados indican que el programa VECTOS implementado en el control rutinario del municipio de Girón fue más costo-efectivo que el programa rutinario de control de vectores en Floridablanca durante el periodo analizado. Con el análisis de costo-efectividad se evaluó el valor económico de un sistema de planeación de programas de enfermedades transmitidas por vectores a nivel de entes territoriales. El modelo basado en la metodología del árbol de decisión permitió determinar que el programa VECTOS implementado en Girón, aparentemente, resultó en una menor carga de dengue en términos de AVAD y a un costo inferior que el programa rutinario de enfermedades transmitidas por vectores en Floridablanca, lo que indicaría que los sistemas basados en la evidencia son más costo-efectivos a la hora de tomar decisiones para el control vectorial en la prevención del dengue, como ya se ha señalado en la literatura científica ^8^.

En cuanto a los costos de los programas evaluados de enfermedades transmitidas por vectores, los resultados coinciden con lo hallado en la literatura especializada en el sentido de que las intervenciones en estas enfermedades implican una cantidad importante de personal remunerado y no remunerado, principalmente, si los programas están enfocados en estrategias que involucran a la comunidad ^17^^-^^19^.

Los diversos estudios de costo-efectividad se han enfocado principalmente en el análisis de intervenciones específicas sobre grupos poblacionales focalizados ^20^^-^^24^. En este sentido, una limitación del presente estudio fue el no haber analizado las estrategias específicas implementadas en cada uno de los municipios, lo que impidió determinar el costo-efectividad a este nivel en población focalizada. La hipótesis que se plantea es que las estrategias implementadas en Girón fueron planeadas y priorizadas de acuerdo con los factores de riesgo entomológicos, epidemiológicos y sociales, suministrados y estratificados por VECTOS en tiempo real y de manera integrada, lo que permitió hacer intervenciones focalizadas por barrio. Esto lleva a concluir que una parte significativa de la efectividad de las estrategias aplicadas se explicaría por el uso de dicho programa.

Los análisis de sensibilidad demostraron que, en situaciones de incertidumbre, la alternativa VECTOS es costo-efectiva frente a los principales parámetros, como la disponibilidad para pagar, el costo de cada uno de los programas y el peso de la discapacidad en el cálculo de los AVAD. El análisis evidenció que la decisión es más sensible a los cambios en el costo de cada programa que a la magnitud de la carga de enfermedad en cada municipio, lo que coincide con la realidad que enfrentan los municipios colombianos que tienen múltiples necesidades de salud y recursos limitados; por lo tanto, es prioritario el uso de análisis como el presentado aquí para optimizar el uso de dichos recursos.

En cuanto a los factores no controlables, el estudio adolece de varias limitaciones. Primero, los múltiples factores implicados en la transmisión de la enfermedad no son considerados por los programas locales de salud pública, que solo se encargan de controlar la población de adultos del vector y sus potenciales criaderos ^25^. Segundo, puede haber subestimación de los casos reportados en ambos municipios. El subregistro comienza con la notificación de los casos y depende de si los casos son reportados en los centros ambulatorios o si son de origen hospitalario, si son confirmados por el laboratorio o son casos sospechosos por síntomas, así como del hecho de si las personas acuden a los puestos de salud en cuanto presentan los síntomas ^26^^,^^27^. Por lo tanto, la estimación de la carga de la enfermedad puede haberse visto afectada en ambos municipios por dicho subregistro, ya que se calculó con los indicadores de morbilidad reportados al Sivigila ^26^.

Si bien en el municipio intervenido con VECTOS la carga de enfermedad fue menor, cabe resaltar que aislar el efecto del programa sobre la morbilidad es complejo, más aún si en periodos anteriores la magnitud de la incidencia del dengue era dispar entre los municipios analizados. Por último, no fue posible elaborar un modelo dinámico que diera una aproximación más realista de la transmisión del dengue en los municipios, y se sabe que esta metodología es más apropiada para el análisis de enfermedades infecciosas ^13^.

En términos de los valores per cápita, los controles rutinarios de ambos municipios fueron comparables al considerar el costo de VECTOS. En el municipio de Girón, el costo per cápita fue de USD$ 0,62, mientras que, en Floridablanca, fue de USD$ 0,66; por lo tanto, el efecto de una escala mayor de costos por cuenta de una mayor población expuesta en el municipio en comparación no se consideró determinante. Aunque el enfoque de este estudio se centró en el costo de la ejecución de los programas de control vectorial, cabe resaltar que, en los de dengue, los costos corresponden a los del sistema de salud y a los indirectos debidos a la pérdida de productividad, lo que indicaría que cada caso evitado tiene un impacto mayor en términos de carga para la sociedad ^28^.

Las implicaciones de los resultados de esta investigación en términos de política, indicarían la necesidad de adoptar el programa VECTOS o una herramienta similar en los entes territoriales, pues mejora la planeación de estrategias y recursos para el control de enfermedades transmitidas por *A. aegypti.*

El programa responde a las necesidades de los entes territoriales que presentan un déficit de personal capacitado para el manejo de los programas de enfermedades transmitidas por vectores ^29^. Los reportes que genera y su interfaz son fáciles de comprender y manejar para facilitar la planeación y optimizar la gestión de los escasos recursos de los programas de enfermedades transmitidas por vectores ^30^. Además, VECTOS permite integrar el componente de vigilancia epidemiológica y entomológica en un sistema de información en tiempo real para la toma de decisiones. La ventaja de este programa es que se ajusta a las políticas locales de enfermedades transmitidas por vectores y las mejora con base en un trabajo coordinado con los entes territoriales, y no en una intervención introducida por un tercero.

Una posible dificultad para el uso de VECTOS es que los municipios deben contar con una dependencia municipal de enfermedades transmitidas por vectores que se encargue de recolectar la información y garantizar su fiabilidad, y que cuente con los recursos suficientes para un plan integral de intervenciones. Dado este contexto, puede decirse que el máximo aprovechamiento del programa se logra en municipios con autonomía fiscal y categoría de población 1, 2 o 3. Para los municipios de categoría 4, 5 y 6, el programa debería ser administrado por el ente departamental debido a la necesidad de contar con los recursos económicos, humanos y tecnológicos necesarios.

Por último, los resultados de este estudio son relevantes en el sentido de que suministran evidencia sobre el costo-efectividad de una tecnología nueva en el control de las enfermedades transmitidas por *A. aegypti*. El marco metodológico empleado puede ser replicado para ampliar el horizonte del estudio y detectar efectos perdurables en el tiempo y en otras localidades, pues el modelo de análisis se alimenta con información disponible en los entes territoriales de Colombia.

### Agradecimientos

A la Secretaría de Salud de San Juan de Girón y su programa de control de enfermedades transmitidas por vectores, y a la Secretaría de Salud de Floridablanca, por el suministro de la información necesaria para este estudio. Al CIDEIM, por su orientación en el manejo de los datos epidemiológicos y la bibliografía sobre las arbovirosis urbanas. Por último, al equipo del Centro de Estudios en Protección Social y Economía de la Salud (PROESA), por el apoyo recibido.
